# The Ecological Conditions That Favor Tool Use and Innovation in Wild Bottlenose Dolphins (*Tursiops* sp.)

**DOI:** 10.1371/journal.pone.0022243

**Published:** 2011-07-20

**Authors:** Eric M. Patterson, Janet Mann

**Affiliations:** Department of Biology, Georgetown University, Washington, D.C., United States of America; Georgia State University, United States of America

## Abstract

Dolphins are well known for their exquisite echolocation abilities, which enable them to detect and discriminate prey species and even locate buried prey. While these skills are widely used during foraging, some dolphins use tools to locate and extract prey. In the only known case of tool use in free-ranging cetaceans, a subset of bottlenose dolphins (*Tursiops* sp.) in Shark Bay, Western Australia habitually employs marine basket sponge tools to locate and ferret prey from the seafloor. While it is clear that sponges protect dolphins' rostra while searching for prey, it is still not known *why* dolphins probe the substrate at all instead of merely echolocating for buried prey as documented at other sites. By ‘sponge foraging’ ourselves, we show that these dolphins target prey that both lack swimbladders and burrow in a rubble-littered substrate. Delphinid echolocation and vision are critical for hunting but less effective on such prey. Consequently, if dolphins are to access this burrowing, swimbladderless prey, they must probe the seafloor and in turn benefit from using protective sponges. We suggest that these tools have allowed sponge foraging dolphins to exploit an empty niche inaccessible to their non-tool-using counterparts. Our study identifies the underlying ecological basis of dolphin tool use and strengthens our understanding of the conditions that favor tool use and innovation in the wild.

## Introduction

Tool use [1], [2] has long been of interest to biologists, anthropologists, and psychologists because of its role in cognition, culture, and hominid evolution [3]–[5]. Studying tool use in animals provides insight into the social, ecological, and evolutionary contexts in which it arises [6]. In mammals and birds, tool use positively correlates with brain size, social transmission, and innovation [7] and is considered to be a sign of cognitive capacity, i.e., problem solving ([8], [9] but see [10], [11]). Most animal tools are used during foraging, especially extractive foraging [1], [12]. In Shark Bay, Western Australia some bottlenose dolphins (*Tursiops* sp.) use marine basket sponge tools to protect their rostra during foraging [13]–[16]. Thus far, we know that sponge foraging, hereafter sponging, is primarily a female behavior, appears to be vertically socially transmitted [14], and is limited to 54 animals (hereafter the spongers) in the eastern gulf of Shark Bay [14]. This solitary behavior occurs in deep channels, requires long dives, and consumes the majority of spongers' activity budgets, yet does not appear to have any fitness costs [14].

**Figure 1:**
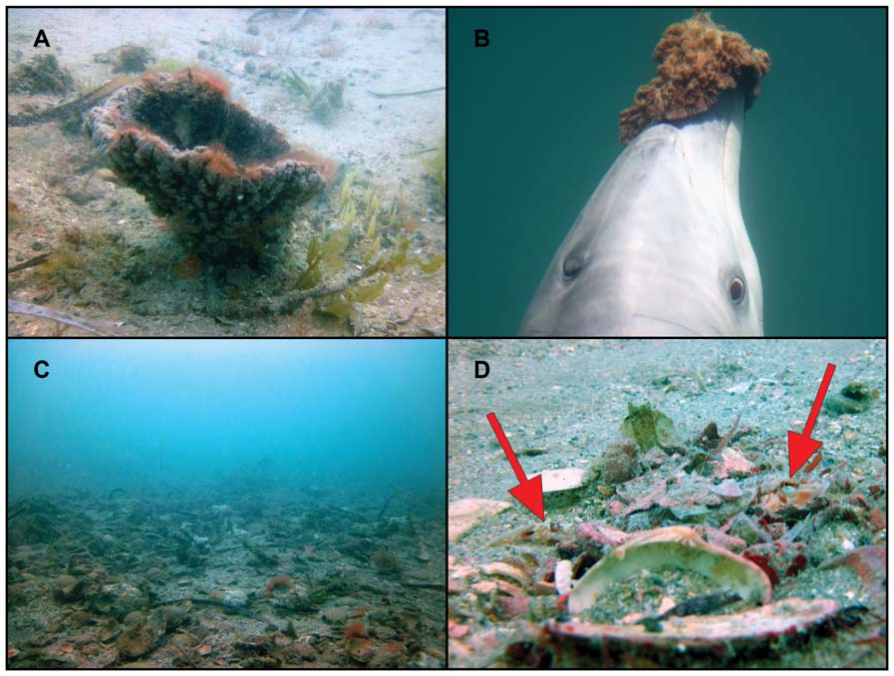
Sponging in Shark Bay. (**A**) marine basket sponge (*Echinodyctium mesenterinum*), (**B**) dolphin wearing a sponge on its rostrum, (**C**) substrate littered with rock, shell, and debris, (**D**) hiding prey, barred sandperch (*Parapercis nebulosa*). All photographs taken by Eric M. Patterson.

On several occasions, during exceptional visibility, researchers have directly observed dolphins *wearing* marine sponges they have removed from the substrate (Figure 1A) over their rostra (Figure 1B) as they probe the rough seafloor (Figure 1C) while searching for hidden prey (Figure 1D). Once prey have been extracted, dolphins drop their sponges, occasionally surface for a quick breath, chase and consume their prey, and finally, return to pick up their sponges and continue foraging [13], [14]. Spongers are suspected to target one or few benthic prey species, including the barred sandperch, *Parapercis nebulosa*, previously mis-identified as *Parapercis clathrata* [14], whose confamilials are consumed by bottlenose dolphins elsewhere [17]. The sponge is thought to function as a shield by providing protection from the sharp and rough seafloor, and possibly venomous or spiny benthic marine organisms, while dolphins search for and extract prey [13], [14]. Dolphins use a single sponge for an average of 68±47 (SD) minutes (Max = 4.4 hrs, Min = 3 minutes, N = 125 sponging bouts) before dropping it to search for a replacement presumably because the sponge has lost its protective value. However, *why* dolphins continuously probe the substrate when searching for prey is unclear given that at other locations (e.g. crater feeding in the Bahamas [12]) dolphins detect buried prey indirectly via echolocation and minimize contact with the seafloor until prey are located. In fact, delphinids' target detection ability using echolocation is quite impressive [18], [19] and has long been used by the U.S. Navy to locate buried mines [20]. So in contrast to other extractive tool users [12], dolphins appear to have the anatomical machinery necessary for the task at hand leaving one to consider: Why do Shark Bay dolphins probe or skim the substrate with their rostra and risk injury instead of simply echolocating for prey?

It is well known that the major acoustic backscatter of fishes (over 90%) comes from the gas-filled swimbladder [21]–[30]. This is not surprising as sound waves most readily echo when encountering a difference in medium density (i.e. liquid to gas) [31]. Fishes without swimbladders have relatively weak acoustic signals, as fish flesh has an acoustic impedance only 10% greater than water [29]. Many fish species have lost their swimbladders, presumably as an adaptation to their benthic or deep-sea lifestyle [32]. While some odontocetes are capable of echolocating swimbladderless prey (cephalopod detection by *Globicephala*, Ziphiidae, and Physeteridae [33], [34]), the majority of these cetaceans' prey are free swimming, not buried [35], and echolocation in these cephalopod specialists appears to be modified by longer click intervals and higher source levels when compared to bottlenose dolphins [36]. We hypothesized that spongers probe the substrate because they target prey that lack swimbladders and thus are difficult to detect with echolocation. Moreover, when these prey are at least partially buried beneath a debris-laden substrate, which causes interfering reverberation and echo clutter (echoes from objects other than the targeted prey) [37], the effectiveness of echolocation is reduced even further. In contrast, dolphins that crater-feed in the Bahamas [38] appear to target buried prey with swimbladders [39] in an uncluttered, soft sand substrate that is less likely to injure dolphins' rostra or dramatically interfere with echolocation [40]. While some echolocation has been documented during sponging, it may only be useful once prey have been extracted and dolphins have dropped their sponges since the sponge itself is likely to interfere with echolocation by obstructing the sound receiving lower jaw and the sound emitting melon [41]. Thus, we predicted that the majority of prey that spongers encounter lack swimbladders.

## Results

**Figure 2:**
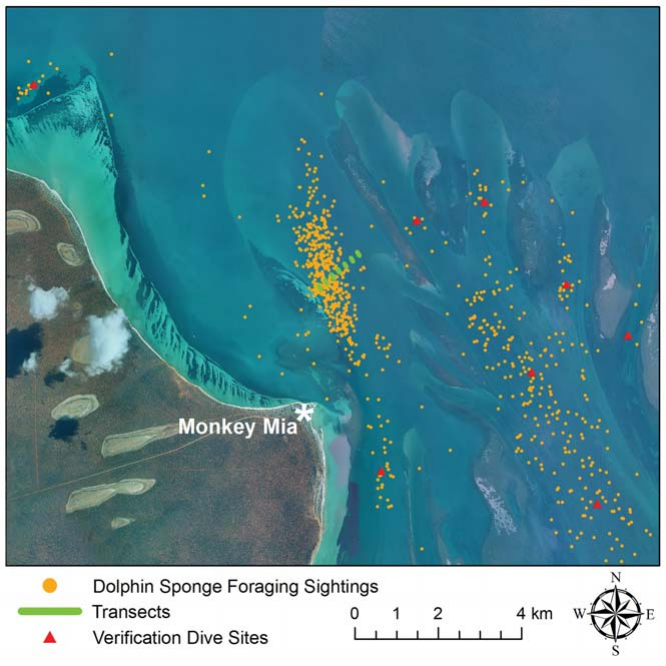
Sponging Map. Boat launch site (Monkey Mia), dolphin sponge foraging sightings, transects, and verification dive sites in Shark Bay, Western Australia.

Of the 134 prey extracted during 13.3 hours of human *Sponging* (Video S1) on both transect and verification dives (Figure 2), 78% lacked swimbladders (Table 1). In contrast only 19% of prey from all *Non-Sponging* dives lacked swimbladders (Table 1). Barred sandperch (Figure 1D), which lack swimbladders, were by far the most common prey extracted during *Sponging*, constituting 65% of the total count, but only made up 18% of *Non-Sponging* prey. Purple tuskfish (*Choerodon cephalotes*), which possess swimbladders, were second in abundance during both *Sponging* and *Non-Sponging* at 17% and 27% respectively; however, 74% of purple tuskfish extracted during *Sponging* were from locations where less sponging has been documented (verification dives, Figure 2), and this species was only extracted when divers probed small seagrass tufts which are both uncharacteristic of channel habitat and less likely to harm dolphins' rostra. Striped whiptail (*Pentapodus vitta*), which possess swimbladders, were the predominant prey during *Non-Sponging* at 34%, but not extracted at all during *Sponging*. No additional families were extracted during *Sponging* on verification dives, although verification dives did yield 3 additional *Non-Sponging* families (Table 1), indicating that our transects are representative of spongers' prey, but not of all non-burrowing prey in the eastern gulf of Shark Bay, which is not surprising.

**Table 1:** Prey abundance from *Sponging* and *Non*-*Sponging*, pooled from both transects and verification dives.

Common Name	Family	Species (if known)	*Sponging* Abundance	*Non-Sponging* Abundance	Swimbladder
barred sandperch	Pinguipedidae	*Parapercis nebulosa*	87	15	N^[53]^ *
sand lizardfish	Synodontidae	*Synodus dermatogenys*	9	0	N^[52]^ *
cuttlefishes	Sepiidae		5	0	N^+^
stingrays	Dasyatidae		1	1	N^+^
lefteye flounders	Bothidae		1	0	N^[32]^ *
painted maskray	Dasyatidae	*Neotrygon leylandi*	1	0	N^+^
tasselsnout flathead	Platycephalidae	*Thysanophrys cirronasa*	1	0	N^[52]^ *
purple tuskfish	Labridae	*Choerodon cephalotes*	23	23	Y^[52,54]^ *
freckled goatfish	Mullidae	*Upeneus tragula*	4	0	Y^[52]^
wrasses	Labridae		2	0	Y^[52,54]^ *
striped whiptail	Nemipteridae	*Pentapodus vitta*	0	29	Y*
margined coralfish	Chaetodontidae	*Chelmon marginalis*	0	6	Y^[52]^ #
blackspot tuskfish	Labridae	*Choerodon schoenleinii*	0	5	Y^[52,54]^ *
bluntheaded wrasse	Labridae	*Thalassoma amblycephalum*	0	2	Y^[52,54]^ *
humpback batfish	Ephippidae	*Platax batavianus*	0	2	Y^[52]^ #
yellowtail clownfish	Pomacentridae	*Amphiprion clarkii*	0	1	Y^[52]^ #
puffers	Tetraodontidae		0	1	Y^[62]^ *

Numbers represent reference(s) used to determine swimbladder status.

*Dissected in this study, +Swimbladder well known to be absent in entire family,

# Additional family encountered on verification dives.

**Figure 3:**
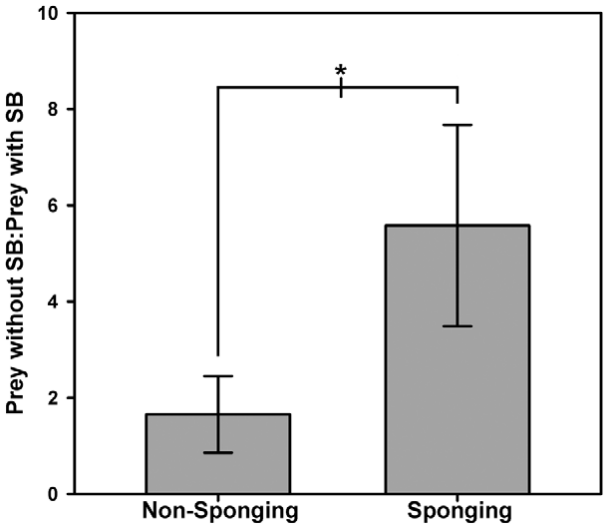
Ratio of prey without swimbladders (SB) to prey with swimbladders during both *Sponging* and *Non-Sponging* on transects. Data were transformed (+1) before ratios were calculated to correct for undefined ratios in samples with zero individuals in either group. Wilcoxon Sign Rank Test W = 28, *P = 0.016.

**Figure 4:**
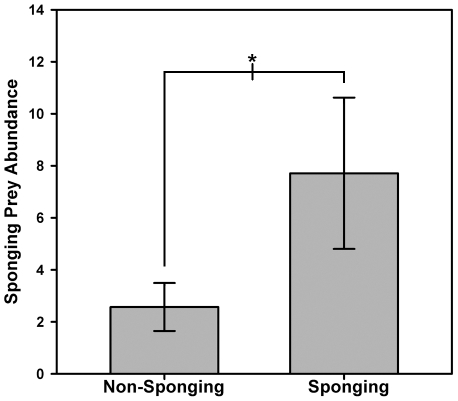
Abundance of prey species extracted during *Sponging* and abundance of these same families during *Non-Sponging* on transects. Wilcoxon sign-rank test W = 28, *P = 0.016.

The ratio of prey *without* swimbladders to those *with* swimbladders was significantly higher during *Sponging* compared to *Non-Sponging* on video transects (Figure 3, Wilcoxon signed-rank test W = 28, P = 0.016), demonstrating that dolphins encounter a greater proportion of swimbladderless prey when sponging than is available to them without disturbing the substrate. Furthermore, the abundance of prey extracted during *Sponging* on transects was significantly greater than that for the same prey families during *Non-Sponging* on transects (Figure 4, Wilcoxon signed-rank test W = 28, P = 0.016), indicating that these prey are primarily concealed in the substrate and that sponging is an effective method of extraction. Together these results show that sponging dolphins extract concealed swimbladderless prey, and do so with greater efficiency than could be done without a sponge tool. Finally, a permutation test revealed that the number of prey families extracted during *Sponging* that lack swimbladders was significantly greater than expected when compared to 27 years of data from the Shark Bay Dolphin Research Project's long-term study (P = 0.0132, Table S1), suggesting that sponging is the primary way dolphins access swimbladderless prey.

## Discussion

Our results demonstrate that sponging dolphins regularly encounter swimbladderless prey that are concealed beneath a rubble-littered substrate. Fish are comprised primarily of water (fish flesh: 82% water, 17% protein, and 0.35% fat by weight [42]) so the density of fish (less the swimbladder) is only slightly greater than seawater (1.076 compared to 1.026 g/cm^3^ [43]). As such, swimbladderless prey have little acoustic impedance and are difficult to detect with echolocation [21]–[30]. To make matters more difficult, Shark Bay channels are strewn with fragmented rock, shell, and coral that are not only likely to injure dolphins' rostra, but also create an echo-cluttering environment leaving dolphins ‘acoustically blind’ to swimbladderless prey. Similarly, echolocating bats seem to have trouble detecting prey on the surface of ponds that are covered with duckweed [44]. Our data demonstrate that spongers have developed a way of effectively extracting hidden prey by probing the substrate with protective sponge tools. Furthermore, when compared to the rest of the Shark Bay population, sponging dolphins appear to specialize in prey that lack swimbladders allowing them to occupy an empty ecological niche.

Alternatively, it is possible that dolphins could listen for soniferous benthic prey and simply use sponges for extraction. In fact, several species of echolocating bats passively listen for prey-generated sounds to detect insects in highly cluttered environments [45]. However, we believe this is unlikely for Shark Bay dolphins since only two prey families that were infrequently extracted during human *Sponging* are reported to be soniferous [46], and both possess swimbladders making them detectable with echolocation anyway. The remaining swimbladderless prey are unlikely to be soniferous since the primary sonic mechanism in fishes is swimbladder movement [46]. Furthermore, fishes mainly produce sound for intraspecific communication [46], not while hidden or buried in the substrate as observed in this study.

The predominant prey extracted during human *Sponging* was the barred sandperch whose behavior was strikingly consistent with dolphin sponging behavior [14]. When barred sandperch were disturbed during human *Sponging*, they swam a few meters away and returned to the substrate often without reburying. This would give dolphins time to drop their sponges and quickly surface to breathe before diving back down in pursuit, as has been regularly documented during our long-term study [14]. New photographs from 2010 of a sponger consuming a small red and brown fish provided further evidence that spongers prey on barred sandperch.

Sponging dolphins may gain several benefits from targeting these prey. First, barred sandperch exhibit consistent, predictable behavior enabling dolphins to employ a single stereotypic sponging behavior. If dolphins extracted a variety of prey species, all having different anti-predator tactics, a uniform sponging behavior would not be as effective. Second, similar to some foods extracted by primate tool users [12], barred sandperch are reliable and can easily and frequently be extracted with a sponge, one every nine minutes during human *Sponging*. However, the average barred sandperch collected was small, only 12.6±4.7 (SD) cm in length (Max = 23 cm, Min = 6 cm, N = 21), which may explain why spongers are more specialized and dedicate more time to foraging than other dolphins in Shark Bay [14]. Finally, extracted foods are often high energy, premium foods [12]. Since fishes with decreased swimbladder volumes typically have increased lipid content [27], the barred sandperch may provide sponging dolphins with an energy-rich meal, similar to some insect larvae extracted by tool-using birds [47]. Accordingly, several barred sandperch have been stored for nutritional analysis. Thus, while requiring more effort than free-swimming prey, barred sandperch likely provide these tool users with a small, yet predictable, reliable, and possibly energy rich food source.

This highly specific tool use has implications for cognition and brain evolution among cetaceans and could even be considered a case of problem solving, a phenomenon difficult to document in the wild, but well established in studies of captive bottlenose dolphins [48]. Our study demonstrates how bottlenose dolphins might use these skills in their natural environment and provides insight into the ecological and evolutionary pressures that promote higher-level cognition. Spongers may have solved the problem of detecting and extracting swimbladderless prey from below a sharp and rough substrate by probing the seafloor with a soft sponge tool. This solution appears to have been adopted at least twice, as unrelated dolphins residing 110 km away in the western gulf of Shark Bay also sponge forage [15]. While this tool use requires sophisticated object manipulation, it appears to provide spongers with equal fitness compared to the rest the population [14].

Due to the difficulty of observing marine fauna, most studies of tool use focus on terrestrial organisms. Using novel underwater techniques, we show that sponge tool-using dolphins target buried prey that lack swimbladders, particularly barred sandperch. Such prey are difficult to detect with echolocation [21]–[30], which, when paired with Shark Bay's cluttered channel substrate, explains why dolphins probe the seafloor and benefit from using sponge tools. Similar to ant-fishing chimpanzees whose tool use is a function of prey type [49], dolphin tool use directly relates to the physical characteristics of their prey. As such, this study emphasizes the critical role ecological factors play in explaining behavioral complexity.

## Materials and Methods

### Ethics Statement

All animal work was approved by the Georgetown University Animal Care and Use Committee (GUACUC) under permits 07-041 and 10-023. Observational and field studies were approved by the Department of Environment and Conservation of Western Australia (DEC) under permits SF007418 and SF006897.

### Study Site

The Shark Bay Dolphin Research Project has an extensive 27-year database that includes demographic, genetic, association, life-history, ecological, and behavioral data on >1,400 dolphins (past and present) residential to a 300 km^2^ area (25°47′S, 113°43′E). Habitat consists of embayment plains (5–13 m), shallow sand flats (0.5–4 m), seagrass beds (0.5–4 m), and bisecting deep channels (7–13 m). One area in particular, just north of Monkey Mia (Figure 2), is extremely well sampled because it is very close to our boat-launching site. As such, spatial patterns of dolphin foraging behavior in this location are unlikely the result of a bias in sampling effort. Using historical data, dolphin-sighting locations were projected using ArcGIS Map 9.3 (WGS 1984 UTM Zone 49S) to determine locations where dolphins sponge forage. Seven semi-permanent transects were established using 1 m long star picket metal posts with attached buoys in locations with the highest numbers of recorded sponging observations. Each 100 m transect was ∼200 m from adjacent transects and further split into two portions (NW and SE) by a mid-stake. The NW portion of each transect was dedicated to systematic observational-video sampling, while the SE portion was designated for prey sample collection.

### Data Collection

For all 7 transects, two certified divers swam out a 50 m tape measure well above the substrate to connect the NW stake to the mid-stake for initial transect setup. After waiting several minutes, both divers then swam back towards the NW stake along one side of the transect line near the substrate and filmed a ∼2 m wide belt transect [50], [51] to determine prey availability near the seafloor without disturbing the substrate (*Non-Sponging*). Next, divers swam back along the other side of the transect (∼2 m to the side of the tape measure) towards the mid-stake with one diver pushing a 2 m long pole with a dead marine sponge attached along the substrate to ferret prey in the same manner as seen by sponging dolphins (*Sponging*), and the other diver filming this human *Sponging* with a Sony HDR-XR500V HD video camera in an AquaticaHD housing (Video S1). All dives were performed on an Airline Supply R360XL Hookah System by J. Sink, and were swam at a consistent speed of ∼17 m min^−1^. On the NW portion of each transect, all prey were simply filmed and allowed to swim away. However, on the SE side of the transects several sample specimens of all species encountered (except Dasyatidae and Sepiidae which all lack swimbladders) were collected using hand nets for identification and dissection. Transect sampling was performed on two different occasions for repeatability, but replicates were averaged to form a single transect value. To confirm that our fine scale study in this well sampled area was representative of greater patterns in the bay, in particular to be sure we had extracted all possible *Sponging* prey species, we also performed verification dives in all other general locations where sponging has been observed (Figure 2). On these verification dives no tape measure was laid, but divers performed and filmed both *Non-Sponging* and *Sponging* as described above. If any new species were encountered, sample specimens were collected for identification and dissection. Only one infrequent prey species was too fast for divers to catch, *Upeneus tragula*, but other species in the same genus are known to have swimbladders [52]. Historical prey species were gathered from the long-term Shark Bay Dolphin Research Project's database and combined with families extracted during *Sponging* to create a total of 29 possible prey families. Families were used instead of individuals due to the potential biases in observing accurate quantities of species consumed (e.g. researchers observe dolphins consuming surface dwelling prey more often than benthic prey since dolphins regularly consume these prey near the surface, in plain site). Using families allows us to avoid these potential biases and explore how dolphins consume the richness of prey they encounter. Swimbladder status for prey families not collected and prey from video only identified to the level of family was determined using primary literature [32], [52]–[62]. All data in Table 1 and Table S1 follow the currently accepted scientific and common names according to Froese and Pauly (2008) [63] and all analyses were performed at the family level.

### Data processing and statistical analysis

Many of the species encountered were quite small, averaging less than 7 cm in length. Such small prey are unlikely to be targeted during sponging because prey this size can easily be obtained at the surface in all habitats, even by young calves [64], [65]. Thus, these prey were removed from prey abundance data. A total of 19 prey encountered could not be identified to the level of family; however, all were estimated to be less than 7 cm in length and thus excluded from the final data set. There is a chance prey were missed during video logging or were simply not captured on film; however, it is likely that all such prey would also be less than 7 cm in length and thus excluded since prey larger than this would be obvious to divers and not overlooked.

The ratio of prey without swimbladders to those with swimbladders was compared between *Sponging* and *Non-Sponging* on transects using a Wilcoxon signed-rank test. Here, prey abundance data were transformed by adding one to all samples before ratios were calculated to correct for samples with zero individuals in either group, which results in an undefined ratio. We also compared the abundance of prey families extracted during *Sponging* on transects to the abundance of these same families during *Non-Sponging* on transects using a Wilcoxon signed-rank test. Finally, we compared data from *Sponging* dives to data from our long-term study. For this final analysis we used a two-tailed permutation test to compare the observed number of families with and without swimbladders from *Sponging* to the expected number based on combined *Sponging* and historical prey data. We re-sampled (with replacement) 8 prey families 10,000 times from 29 possible families (Table S1) and determined the likelihood of obtaining our observed human *Sponging* data by chance. While Table 1 present abundance data pooled from all dives for descriptive purposes, all statistical analyses were performed only using the systematic transect data for which we could be sure that the substrate traversed during *Sponging* and *Non-Sponging* were equal. All statistical tests were performed in R 2.12.1 statistical environment (R Development Core Team, 2011) and considered significant for P<0.05.

## Supporting Information

Table S1 *Sponging* and historical prey families. Numbers represent reference(s) used to determine swimbladder status. *Dissected in this study, +Swimbladder well known to be absent in entire family.
(DOCX)

Video S1 Divers performing human *Sponging*. Prey in order of appearance: *Sepia* sp., *Parapercis nebulosa*, *Parapercis nebulosa*, *Neotrygon leylandi*, *Parapercis nebulosa*, *Parapercis nebulosa*, *Parapercis nebulosa*, *Synodus dermatogenys*.
(MP4)
