# Correction: The Ecological Conditions That Favor Tool Use and Innovation in Wild Bottlenose Dolphins (*Tursiops* sp.)

**DOI:** 10.1371/annotation/2555a3f6-117f-42e2-b89c-c568bd6618c9

**Published:** 2011-08-05

**Authors:** Eric M. Patterson, Janet Mann

The following funders provided living stipends for author Eric Patterson:

Achievement Rewards for College Scientists: http://www.arcsfoundation.org/

Office of Naval Research Grant #10230702: http://www.onr.navy.mil/

