# Supplementary material for: The Ecological Conditions That Favor Tool Use and Innovation in Wild Bottlenose Dolphins (Tursiops sp.)
Source: PLoS One. 2011 Jul 20;6(7):e22243. doi: 10.1371/journal.pone.0022243 (PMC3140497; doi:10.1371/journal.pone.0022243)
Supplement: Table S1 — Sponging and historical prey families. Numbers represent reference(s) used to determine swimbladder status. *Dissected in this study, +Swimbladder well known to be absent in entire family. (DOCX) [file pone.0022243.s001.docx]

| **Common Name** | **Family** | **Extracted During *Sponging*** | **Swimbladder** |
| --- | --- | --- | --- |
| lefteye flounders | Bothidae | Y | N^[^[^32^](#_ENREF_32)^]*^ |
| stingrays | Dasyatidae | Y | N^+^ |
| sandperches | Pinguipedidae | Y | N^[^[^53^](#_ENREF_53)^]*^ |
| flatheads | Platycephalidae | Y | N^[^[^52^](#_ENREF_52)^]*^ |
| cuttlefishes | Sepiidae | Y | N^+^ |
| lizardfishes | Synodontidae | Y | N^[^[^52^](#_ENREF_52)^]*^ |
| octopuses | unidentified Octopoda families | N | N^+^ |
| squids | unidentified Teuthida families | N | N^+^ |
| wrasses | Labridae | Y | Y^[^[^52^](#_ENREF_52)^,^[^54^](#_ENREF_54)^]*^ |
| goatfishes | Mullidae | Y | Y^[^[^52^](#_ENREF_52)^]^ |
| bonefishes | Albulidae | N | Y^[^[^52^](#_ENREF_52)^]^ |
| needlefishes | Belonidae | N | Y^[^[^52^](#_ENREF_52)^]^ |
| jacks and pompanos | Carangidae | N | Y^[^[^52^](#_ENREF_52)^,^[^55^](#_ENREF_55)^]^ |
| herrings, shads, sardines, menhadens | Clupeidae | N | Y^[^[^52^](#_ENREF_52)^,^[^56^](#_ENREF_56)^]^ |
| halfbeaks | Hemiramphidae | N | Y^[^[^52^](#_ENREF_52)^,^[^57^](#_ENREF_57)^]^ |
| filefishes | Monacanthidae | N | Y^[^[^52^](#_ENREF_52)^,^[^56^](#_ENREF_56)^]^ |
| mullets | Mugilidae | N | Y^[^[^52^](#_ENREF_52)^]*^ |
| snake eels | Ophichthidae | N | Y^[^[^52^](#_ENREF_52)^,^[^58^](#_ENREF_58)^]^ |
| eeltail catfishes | Plotosidae | N | Y^[^[^52^](#_ENREF_52)^]^ |
| bluefishes | Pomatomidae | N | Y^[^[^52^](#_ENREF_52)^,^[^56^](#_ENREF_56)^]^ |
| dottybacks | Pseudochromidae | N | Y^[^[^52^](#_ENREF_52)^]^ |
| scats | Scatophagidae | N | Y^[^[^52^](#_ENREF_52)^]^ |
| tuna, mackerels, and bonitos | Scombridae | N | Y^[^[^52^](#_ENREF_52)^]^ |
| sea basses | Serranidae | N | Y^[^[^52^](#_ENREF_52)^]^ |
| rabbitfishes | Siganidae | N | Y^*^ |
| smelt-whitings | Sillaginidae | N | Y^[^[^52^](#_ENREF_52)^,^[^60^](#_ENREF_60)^,^[^61^](#_ENREF_61)^]^ |
| porgies | Sparidae | N | Y^[^[^52^](#_ENREF_52)^]^ |
| grunters or tigerperches | Terapontidae | N | Y^[^[^52^](#_ENREF_52)^]^ |
| puffers | Tetraodontidae | N | Y^[^[^62^](#_ENREF_62)^]*^ |
